# Fibrinolysis and Inflammation in Venous Thrombus Resolution

**DOI:** 10.3389/fimmu.2019.01348

**Published:** 2019-06-14

**Authors:** Subhradip Mukhopadhyay, Tierra A. Johnson, Nadire Duru, Marguerite S. Buzza, Nisha R. Pawar, Rajabrata Sarkar, Toni M. Antalis

**Affiliations:** ^1^Center for Vascular and Inflammatory Diseases, University of Maryland School of Medicine, Baltimore, MD, United States; ^2^Department of Surgery, University of Maryland School of Medicine, Baltimore, MD, United States; ^3^University of Maryland Marlene and Stewart Greenebaum Comprehensive Cancer Center, University of Maryland School of Medicine, Baltimore, MD, United States; ^4^Department of Physiology, University of Maryland School of Medicine, Baltimore, MD, United States

**Keywords:** venous thromboembolism, venous thrombus resolution, DVT, PE, inflammation, fibrinolysis, plasminogen, innate immunity

## Abstract

Clinical observations and accumulating laboratory evidence support a complex interplay between coagulation, inflammation, innate immunity and fibrinolysis in venous thromboembolism (VTE). VTE, which includes deep vein thrombosis (DVT) and pulmonary embolism (PE), and the subsequent complications of post-thrombotic syndrome (PTS), are significant causes of morbidity and mortality in patients. Clinical risk factors for VTE include cancer, major trauma, surgery, sepsis, inflammatory bowel disease, paralysis, prolonged periods of immobility, and aging. Abnormalities in venous blood flow or stasis initiates the activation of endothelial cells, and in concert with platelets, neutrophils and monocytes, propagates VTE in an intact vein. In addition, inflammatory cells play crucial roles in thrombus recanalization and restoration of blood flow via fibrinolysis and vascular remodeling. Faster resolution of the thrombus is key for improved disease prognosis. While in the clinical setting, anticoagulation therapy is successful in preventing propagation of venous thrombi, current therapies are not designed to inhibit inflammation, which can lead to the development of PTS. Animal models of DVT have provided many insights into the molecular and cellular mechanisms involved in the formation, propagation, and resolution of venous thrombi as well as the roles of key components of the fibrinolytic system in these processes. Here, we review the recent advances in our understanding of fibrinolysis and inflammation in the resolution of VTE.

Cardiovascular diseases, involving disorders of the heart and blood vessels, are a leading cause of death and disability globally. Thrombosis is the major underlying cause of the pathology of the three major cardiovascular disorders: ischemic heart disease (acute coronary syndrome), stroke and venous thromboembolism (VTE) (1). Historically, cardiovascular diseases were believed to be solely caused by aberrations in the structures and functions of the cardiovascular system, but in recent years, the role of systemic inflammation as well as the involvement of innate and adaptive immunity in the pathophysiology of cardiovascular diseases has become clear (2–5).

VTE, which includes deep vein thrombosis (DVT) and pulmonary embolism (PE), is an exceedingly common and serious clinical problem (6–8). DVT occurs when a thrombus forms in a vein, usually in the deep veins of the legs or pelvis. The most serious complication of DVT occurs when part of the clot detaches and travels via the circulation to the pulmonary arteries, causing a blockage or pulmonary embolism (PE). PE can be fatal due to hypoxia and circulatory collapse (9). Initiation of the formation of an intravascular venous thrombus involves a complex interplay between innate immune cells, platelets, and the venous endothelial cells (10). The activation of the coagulation cascade by these cells and the deposition of fibrin leads to the formation of the venous thrombus. Conversely, the immune cells involved in the initiation of the blood clot formation also express and release fibrinolytic factors and thus orchestrate the resolution of the venous thrombus by modulation of the fibrinolysis system. These concepts are illustrated in Figure 1. This review summarizes the recent advances in our understanding of the interplay between inflammation, innate immunity and fibrinolysis focusing on venous thrombosis and its resolution.

**Figure 1:**
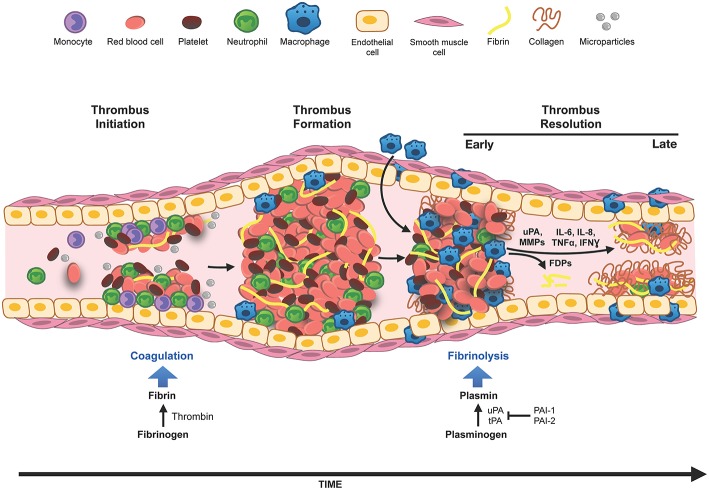
Innate immune cells in DVT. Venous thrombosis can be initiated by venous stasis, increased blood hypercoagulability or endothelial damage. Innate immune cells, neutrophils, and monocytes, bind to the activated vascular endothelium and along with platelets, initiate thrombus formation and fibrin deposition. The thrombus grows by deposition of more fibrin, accumulation of red blood cells and immune cells. Thrombus infiltrating neutrophils and macrophages (differentiated from monocytes) modulate generation of plasmin and matrix metalloproteinases (MMPs), and thus set the stage for fibrinolysis and the collagen remodeling required for the resolution of the thrombus. In the early phase of thrombus resolution, fibrinolysis occurs at a high rate generating fibrin degradation products (FDPs), intrathrombus collagen fibrils start to appear, and thrombus-associated immune cells are induced to produce inflammatory cytokines and various proteases. As the thrombus matures, the rate of fibrinolysis slows down, intrathrombus collagen deposition increases, matrix remodeling via macrophage secreted MMPs occurs and eventually blood flow through the thrombus is restored. Resolution of inflammation and acceleration of this process is believed to be beneficial for restoring vein wall patency and reducing the pathology associated with PTS.

## Causes of VTE

VTE is a multifactorial process and is associated with several different risk factors. The prevalence of these risk factors predisposes an individual to venous thromboembolic events. In 1856, the German physician Rudolf Virchow first postulated that VTE was caused by abnormalities in the normal blood flow or stasis, increased blood hypercoagulability and endothelial damage or dysfunction, which later came to be known as “Virchow's triad.” Reduced blood flow caused by prolonged periods of inactivity, especially in elderly subjects, long hospitalizations due to illness, pregnancy and long distance travel with limited movement such as air-travel, are associated with increased risk of VTE (11). Similarly, individuals with increased levels of clotting factors in the circulation, resulting from diseases, medications, or inherited traits, have increased risk of VTE (12). Tumor cells frequently produce large amounts of the procoagulant transmembrane receptor tissue factor, which can be released in tumor-derived microparticles rendering the blood hypercoagulable in individuals with cancer, and is likely to be a major cause for the observed high incidence of VTE in cancer patients (13). In fact, cancer patients make up 20% of all newly diagnosed VTE (14). Finally, trauma or damage to the venous endothelium can lead to disturbances in the balance between procoagulant and anticoagulant properties of the venous endothelium and are also predisposing factors for VTE (15).

Patients present with either acute DVT wherein the clot has been present for <14 days, or chronic DVT, when the clot is present for more than 28 days and sometimes indefinitely (16, 17). Anticoagulant therapy is used to prevent the formation of more clots and prevents thrombus propagation. In cases of severe, life-threatening PE, treatment also consists of thrombolytic therapy (streptokinase, urokinase, or tissue plasminogen activator) and catheter directed or surgical thrombectomy to remove the thrombus (18).

## Post-Thrombotic Syndrome (PTS)

One of the major complications of chronic DVT is the development of post-thrombotic syndrome (PTS) (19). PTS is a debilitating condition with symptoms including difficulty in walking, leg swelling and ulceration in the skin of the affected leg. PTS occurs in about one in 2–3 patients who had an earlier episode of thromboembolism (20, 21). In 10% of the patients suffering from PTS the symptoms become severe (20, 22). When a thrombus forms, a natural inflammatory response is initiated, mediated by the immune cells present in the thrombus, that ultimately leads to reabsorption of the clot through fibrinolysis and thrombus recanalization, or the restoration of blood flow (23). While the inflammatory response is necessary for the contraction and recanalization of the thrombus, the very presence of this inflammation causes damage to the surrounding vein wall and the venous valves leading to valvular dysfunction (24). Failure to recanalize the thrombus and the ensuing obstruction of blood flow can cause venous hypertension below the level of the obstruction, resulting in venous reflux, which is a major cause for the development of PTS (22, 24). Venous reflux can also result from the entrapment or destruction of the delicate venous valve leaflets by the resolving thrombus. The involvement of inflammation in the development of PTS is supported by the observation that VTE patients with PTS have higher circulating levels of the inflammatory markers, IL-6 and ICAM-1, compared to patients without PTS (25). Over 30% of patients with DVT develop chronic venous insufficiency (26), and patients with thrombi that fail to recanalize within the first 6 months from the occurrence of DVT have a higher likelihood of developing PTS (27, 28). Clinical studies suggest that vein wall changes occur as a direct consequence of initial thrombus burden (29). A more rapid resolution of the thrombus is thus beneficial to the preservation of the vein wall patency and valvular function (30, 31).

## Understanding DVT and its Resolution—Animal Models

Our current understanding of the molecular mechanisms involved in DVT and its resolution is largely derived from the use of rodent models of stasis- or stenosis-induced venous thrombosis, where the inferior vena cava of the animal is either completely or partially ligated to induce formation of a venous thrombus (32–37). These animal models mimic many of the clinical and pathophysiological features observed in human DVT (34, 38), including the presence of inflammatory cells in the milieu (10) (Figure 1) and the complex interactions of the thrombus with the vein wall which mimic the biomechanical compliance changes seen in patients with PTS (39, 40). Like human DVT, the formed venous thrombi are fibrin and red blood cell rich, and have a laminar structure consisting of layers of platelets, leukocytes, and fibrin, that encompass the main erythrocyte mass (41). They differ from arterial thrombi in being platelet poor and red blood cell rich. Of the different experimental animal models, murine models offer the distinct advantage of genetic manipulation to dissect molecular mechanisms, which has proven very useful in providing insights into the cellular and molecular processes involved in human DVT. However, these models also have limitations. A recent consensus endorsed by the International Society on Thrombosis and Hemostasis, and the ATVB Council of the American Heart Association provides a useful guide for the application of murine models to VTE research (37). Below are highlighted the most frequently used models:

### IVC Stasis Model

**Figure 2:**
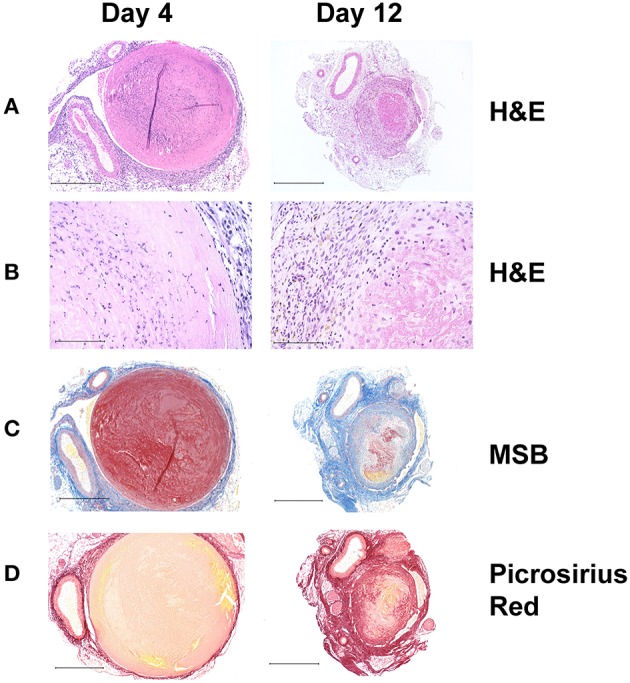
Histochemical analysis of thrombus sections from a stasis induced mouse model of DVT. In this model, thrombus formation occurs maximally at day 4 and resolves naturally thereafter with day 12 serving as a measure of thrombus resolution. **(A)** Hematoxylin and Eosin (HandE) stain showing overall tissue morphology; (Original magnification x100, Scale bar 500 μm) and **(B)** cell infiltrates. The nucleated cellular population at day 4 comprises mostly of neutrophils and few macrophages, whereas both macrophages and neutrophils can be seen at day 12 (Original magnification x400, Scale bar 100 μm). **(C)** Martius Scarlet Blue (MSB) stain showing fibrin content in red (Original magnification x100, Scale bar 500 μm). **(D)** Picrosirius Red stain showing collagen content in red (Original magnification x100, Scale bar 500 μm). As the thrombus resolves, it becomes smaller in size, fibrin content is decreased via fibrinolysis and there is an increase in intrathrombus collagen content.

This is a robust model that accurately mimics many features of human DVT and it is well-established in the DVT literature (42–47). Stasis is induced by complete ligation of the inferior vena cava immediately below the renal veins, and all the side branches are either cauterized or ligated (48). The model produces thrombi of reproducible size and variation between animals is relatively small (Figure 2). While the model does not reproduce the clinical scenario where a thrombus is non-occlusive, it does reproducibly mimic complete occlusion, which is pathologically significant since human acute DVTs are initially occlusive in 88% of cases (49). A limitation of this model is that the lack of flow limits the effect of systemically administered agents on the thrombus.

### IVC Stenosis Model

This model also involves IVC ligation, except that the thrombus grows in the presence of blood flow, mimicking partial occlusion of the vein in clinical scenario and represents a chronic DVT condition. In this model, a spacer (either a small gauge needle or suture) is placed on the IVC before the ligation and is removed after the ligation is performed to allow for very low blood flow through the vena cava (10, 50–53). One serious limitation of this model is the large variation in thrombus size after ligation and absence of a thrombus in a significant number of animals. A variation of this model that includes endothelial damage created by placement of a vascular clip onto the IVC has also been reported (54, 55).

### Ferric Chloride-Induced Venous Thrombosis Model

In this model, venous thrombus formation is initiated by oxidative damage to the vein wall by using a ferric chloride solution (56). A filter paper soaked ferric chloride is placed on the vein, such as femoral vein or inferior vena cava, for a period of time and upon removal of the filter paper, a thrombus is formed that represents an acute complete occlusive venous thrombus. A major drawback of this model is that it mimics only a small percentage of human DVT cases where the cause of DVT is due to endothelial damage, such as in cases of trauma or burn injury.

### Electrolytic IVC Thrombosis Model

This model involves initiation of a venous thrombus by electrical stimulation of the vena cava endothelium using an electrical impulse (57). Major advantages of this model are that the thrombus is formed in the presence of blood flow and is relatively consistent in size. Prolonged time to induce the thrombus is a major drawback of this model.

### Pulmonary Embolism Models

While much has been gained from animal models of VTE, these models fail to reproduce the sequence of both VTE and PE. Specific murine PE models have been developed to study the effects of either gene deletions or specific pharmacological manipulations on the outcome of PE. Many of these models involve intravenous administration of various coagulation factors, such as thrombin (58, 59), thromboplastin (60), or collagen (61) via either the inferior vena cava, jugular vein or tail vein, resulting in rapid onset of widespread thrombosis at the pulmonary level. A photochemical PE model that has been used employs direct irritation of the venous endothelium by use of the photosensitizing dye, Rose Bengal (tetrachlorotetraiodofluorescein), which generates oxygen radicals and focal vascular injury after exposure to green light (62). A novel model of direct quantification of PE events following femoral VTE induced by ferric chloride has also been described in which PE burden is detected by fluorescent labeling of platelets and *in vivo* quantification of emboli in pulmonary arteries (63).

## Formation of Venous Thrombi

Thrombus formation generally starts at the venous valve sinuses, the slowing down of the blood flow around the valvular sinuses and the consequent rise in the local hematocrit value, naturally predisposes those areas to the event of thrombosis (64). This is supported by the clinical observation that in most of the lower extremity DVT cases, thrombus formation starts in the soleal veins of the calf and then propagates to other veins (65, 66). In microscopic examination of small thrombi formed in the valve pockets from human patients, two major regions can be seen: red areas, near to the valve pockets that are rich in red blood cells and fibrin, and white areas comprising mostly of platelets (67). In contrast to venous thrombosis, arterial thrombosis is initiated after an atherosclerotic plaque rupture and arterial thrombi are rich in platelets and white in appearance. The presence of a high number of red blood cells in a fresh venous thrombus was previously believed to be result of passive trapping of the red blood cells in a growing fibrin meshwork; however, recent data suggests that this may be a coordinated process involving specific interactions between red blood cells and different components in the milieu of the thrombus. Red blood cells can interact with both platelets and leukocytes via integrin mediated interactions (68, 69). In a mouse model of ferric chloride-induced arterial thrombosis, it was shown that red blood cells were the first type of cells to arrive and bind to the endothelium at the site of thrombus initiation (70). Subsequent interaction of the endothelium bound red blood cells with platelets involving glycoprotein Ib-α receptor was required for the thrombus propagation. A similar mechanism is also possible in case of venous thrombosis.

Venous thrombus formation is initiated by the activation of the coagulation cascade, followed by thrombin-induced conversion of fibrinogen to fibrin (71). The risk of VTE is associated with elevation in the blood fibrinogen level (hyperfibrinogenemia) as well as abnormal fibrin clot structure and function. When compared with individuals with normal circulating fibrinogen levels, individuals with higher fibrinogen levels (>4 g/L) were 2-fold more disposed to experience VTE and this was significant in older patients (72). This finding was also validated in a rodent model, where intravenous infusion of fibrinogen in mice resulted in a shorter time to vessel occlusion and a larger thrombus (73). On the other hand, genetic mutations that lead to defects in fibrin function and quantity in the circulation are also associated with increased incidences of VTE. Afibrinogenemia (absence of fibrinogen) and hypofibrinogenemia (low plasma level), as well as dysfibrinogenemia (normal level but altered function) conditions are also known to be at higher risk for VTE events (74). Further, there are reports of altered fibrin clot structure in patients with idiopathic thromboembolism that appeared to have a genetic component (75).

The interaction of blood leukocytes with the activated venous endothelium is a major event in venous thrombus formation. The release of tissue factor from endothelium-bound monocytes and leukocyte microparticles initiates the coagulation cascade (10), leading to the activation of thrombin and the conversion of fibrinogen to fibrin. This is also associated with changes in the cytokine milieu within the thrombus, originating from the interactions between the activated platelets, red blood cells, leukocytes and the endothelium (Figure 1). These ensuing inflammatory signals augment thrombus formation and initiate the eventual process of thrombus resolution (76, 77). As blood flows over the growing thrombus, fibrin and various cells are deposited in alternating layers, giving rise alternating white and red bands typical of Lines of Zahn (78). While platelet numbers are lower than in arterial thrombi in the rapidly growing venous thrombi, activated platelets also express P-selectin, which aids in the infiltration of peripheral leukocytes into the thrombus. Neutrophils are among the first leukocytes to be recruited to the thrombus, followed by monocytes that differentiate into macrophages (79). In the acute or early phase of thrombus formation, the fibrin network undergoes rapid polymerization. Extracellular DNA fibers, released by neutrophils during inflammation and known as neutrophil extracellular traps (NETs), stimulate thrombus formation and coagulation (Figure 1) and are abundant in thrombi in animal models of DVT (51).

## Venous Thrombus Resolution

As the thrombus ages, leukocyte infiltration into the thrombus increases and the thrombus appears more structured with the deposition of collagen fibrils (Figure 1). Resolution of the thrombus involves both neutrophils and monocytes that are capable of modulating the generation and activity of plasmin, required for fibrinolysis, or the degradation of the fibrin network. These cells also secrete matrix metalloproteases (MMPs) (80–83), which can further activate the plasminogen activation system and set the stage for the degradation and remodeling of extracellular matrix components in the more mature thrombus (Figure 1). It is believed that thrombus-associated fibroblasts deposit collagen after activation by TGF-beta, although direct evidence supporting this is still lacking. Maturation of the thrombus is marked by a decrease in the overall fibrinolytic activity within the thrombus (84). In clinical cases, the majority of the patients have reduced D-dimer level present in the circulation 1 month after the first episode of DVT (85). Neutrophils and monocytes continue to secrete MMPs as well as various inflammatory cytokines, both of which contribute to the remodeling and resolution of the thrombus.

In the late phase of thrombus resolution, differentiated macrophages infiltrate the thrombus and endothelial cell lined channels within the thrombus also become apparent. Distinct layers of collagen deposition starting at the vein wall adjacent area and protruding toward the center of the thrombus become readily visible (Figure 2D, right panel). Macrophages continue to secrete MMPs (86), required for degradation and remodeling of the collagen matrix. It has been demonstrated that endocytosis of collagen molecules by CD206 positive macrophages is the major route of collagen turnover *in vivo* (87), although whether the same phenomenon occurs in a venous thrombus remains to be investigated.

## The Role of Fibrin(Ogen)

Fibrin and its degradation products themselves are known for modulating inflammatory responses in variety of immune cells. Fibrin can enhance leukocyte migration to the deposition site (88, 89). Direct interaction of fibrin via CD11b/CD18 integrin with peripheral blood mononuclear cells results in heightened production of inflammatory cytokines such as TNF-alpha, IL-6 and IL-1beta (90, 91). Studies have shown interaction of fibrin with RAW 264.7 macrophages results in enhanced production of macrophage inflammatory protein-1alpha (MIP-1alpha), MIP-1beta, MIP-2, and monocyte chemoattractant protein-1 (92). It was postulated that this interaction is mediated by the Toll-like receptor (TLR)-4, since the response was abrogated *in vivo* in mice that express mutant TLR-4 (92). On the other hand, studies with fibrinogen-γ^390−396A^ knock-in mice identified the CD11b/CD18 integrin as the primary receptor for the fibrin mediated pro-inflammatory macrophage cytokine secretion (93). In addition, interaction of fibrin with endothelial cells results in induction of IL-8 mRNA (94), whereas fibrin enhances binding of leukocytes to the vascular endothelium via ICAM-1 (95). Functional studies have shown that fibrinogen gene deletion or pharmacological depletion of fibrin reduced inflammation and delayed the onset of multiple sclerosis in animal models (96), indicating a role for fibrin in the modulation of inflammatory responses. While there is no direct evidence for fibrin modulation of inflammation in DVT, the presence of fibrin is likely to augment pro-inflammatory responses and fibrin-initiated modulation of the inflammatory cascade in the thrombus milieu cannot be ruled out.

## Fibrinolysis and Venous Thrombus Resolution

Activation of the inactive zymogen plasminogen to the serine protease plasmin, which digests the fibrin component of a thrombus, is the key step in fibrinolysis and thrombolysis. Plasmin is an essential element of early venous thrombus resolution, contributing not only to fibrinolysis, but also leukocyte infiltration, the activation of other protease zymogens (e.g., MMP-9) and the regulation of coagulation factors (97–101). Plasminogen is synthesized in the liver and circulates in the blood, wherein it becomes incorporated into the thrombus as it is forming due to its affinity for lysine residues on fibrin (102). The degradation of fibrin polymers by plasmin results in release of fragment E and two molecules of fragment D which are released as a covalently linked dimer (D-dimer) (103). Detection of D-dimer in the circulation is a marker of ongoing clot formation and an elevated D-dimer level in patients after treatment for DVT predicts an ongoing risk of recurrent VTE (85, 104). It should be noted that due to lack of D-dimer standardization, elevated levels of D-dimer are usually followed up by additional screenings to confirm the presence of ongoing VTE events.

The conversion of plasminogen into plasmin is primarily mediated by two plasminogen activators, tissue-type (tPA) and urokinase-type plasminogen activator (uPA), which proteolytically cleave between residues Arg^561^-Val^562^ of plasminogen, inducing its activation (105). Whereas, tPA is primarily involved in clot dissolution (106), uPA principally regulates plasmin-mediated cell migration and tissue remodeling, as well as the activation of latent growth factors and cytokines (107). tPA is slowly released from endothelial cells to affect activation of endogenous plasminogen (108) and this activity is accelerated when in a ternary complex with fibrin (109). Additional plasminogen-cleaving serine proteases include several coagulation proteins and plasmin itself [reviewed in (102)].

Several published gene targeting and gene transfer studies have confirmed the significant role of tPA-mediated plasminogen activation in removing fibrin from the vascular tree and maintaining vascular patency (110), but also established a less appreciated role of uPA in prevention of thrombosis during traumatic or inflammatory conditions (111). uPA and its cellular receptor, uPAR, are produced by macrophages, and these cells have proved to be critical components of the process of thrombus resolution because they are known to produce a variety of proteases, growth factors, chemokines and matrix-degrading enzymes (55). While tPA has been implicated in the resolution of human DVT (106), genetic deficiency of tPA in mice did not affect thrombus resolution. On the other hand, uPA deficiency markedly impairs thrombus resolution in mice (55). Further, it has been shown by bone marrow transplantation studies that uPA derived from bone marrow cells was responsible for venous thrombus resolution (55). Indeed, when uPA is delivered to formed venous thrombi in mice, either by direct injection into the thrombi or via transducing macrophages, resolution occurs more rapidly (112, 113), demonstrating its critical role in this process.

## Inhibition of Fibrinolysis

The fibrinolytic system is tightly regulated and is normally restricted in the thrombus (101). Major inhibitory regulators of fibrinolysis are members of the family of serine protease inhibitors, known as serpins (114). In the circulation, plasmin binds rapidly to the serpin α_2_-antiplasmin (a2AP, also known as SERPINF2) and is thereby inactivated [reviewed (115)]. In the thrombus, the interaction of plasmin with a2AP is blocked because the lysine-binding sites and the catalytic site of plasmin are occupied by fibrin, suggesting that that the primary role of a2AP is not to regulate plasmin-mediated fibrinolysis, but to inhibit circulating plasmin in order to prevent activation of fibrinogen (116). Crosslinking of a2AP to fibrin also significantly enhances the resistance of fibrin to degradation by plasmin through competitive inhibition (117, 118). Congenital deficiency of a2AP causes a rare bleeding disorder because of increased fibrinolysis (119). Deficiency of a2AP in mice resulted in decreased mortality in a photochemical injury model of PE (62), supporting the importance of plasmin activity in acute PE.

The activities of the plasminogen activators must also be tightly controlled and many studies demonstrate that the serpin plasminogen activator inhibitor-1 (PAI-1; also known as SERPINE1) is the primary inhibitor of both tPA and uPA induced fibrinolysis (120). PAI-1 is secreted from liver and is synthesized by a variety of cell types including, hepatocytes, platelets, vascular endothelium, adipose tissue, monocytes and macrophages (102, 121). Thrombolysis resistance is linked to the PAI-1 secreted from the alpha-granules of activated platelets (122). Measurements of PAI-1 levels in 25 venous thrombi and 21 arterial thrombi showed an inverse correlation between the PAI-1 levels and resistance to thrombolysis (123).

PAI-1 circulates in plasma and numerous studies associate the increased levels of PAI-1 activity with reduced fibrinolytic responses in patients with DVT (124). A unique feature of PAI-1 is its lack of disulfide bonds, allowing it to circulate in plasma in three forms: active, inactive and latent (125). The latent form can be stabilized by vitronectin binding (126). It has been considered that elevated PAI-1 could suppress fibrinolysis and increase thrombosis, hence increasing the clinical manifestations of DVT. However, studies on the role of elevated levels of PAI-1 in patients with venous thrombosis have been contradictory (127, 128). A 4G polymorphism located in the promoter region of the PAI-1 gene has been reported to be associated with elevated levels of PAI-1 and further was correlated with risk of DVT (129, 130). Further, it has also been reported that preoperative plasma PAI-1 is an independent risk factor for the onset of DVT in patients who went through total hip arthroplasty (131). In contrast, a study involving 308 individuals who developed VTE and 640 controls showed no association between the plasma levels of fibrinolytic factors, including PAI-1 antigen, and VTE (132). PAI-1 inhibitors have been suggested to be used against the development of intravascular thrombosis, however preclinical animal studies using PAI-1 inhibitors to decrease circulating PAI-1 levels have yielded both negative and positive results [reviewed in (133)].

PAI-1 is a major inhibitor of plasma fibrinolytic activity. Overexpression of PAI-1 in transgenic mice results in increased cellular fibrin and platelet rich occlusions in the tail and hindlegs (134). Conversely, mice genetically deficient in PAI-1 possess induces a mild hyperfibrinolytic state and accelerated clot lysis compared to wild type mice (120). In addition, PAI-1 deficient mice show a greater resistance to venous thrombosis after local injection of endotoxin in the footpad and increased capacity to lyse experimental plasma clots in a PE model (120). Several groups have investigated the role of PAI-1 in VTE using mouse models and found that PAI-1 plays a role in both venous thrombus formation and resolution, highlighting the importance of the balance of prothrombotic and antithrombotic activities in DVT. PAI-1 deficiency through pharmacological inhibition or genetic deletion, results in delayed total venous occlusion (135, 136) and decreased early thrombus size (137, 138). In a stasis IVC ligation model, mice with genetic deletion of PAI-1 resulted in a significant improvement in venous thrombus resolution with also a significant increase in the vein wall fibrosis (100). This has also been shown by Siefert et al. wherein PAI-1 deficiency results partially impaired venous thrombus formation and accelerated venous thrombus resolution (46), demonstrating that PAI-1 influences both processes. Conversely, PAI-1 overexpression in mice had larger venous thrombosis, but they also had reduced vein wall fibrosis (139).

In addition to PAI-1, the serpin plasminogen activator inhibitor type-2 (PAI-2, also known as SERPINB2), regulates plasminogen activation in models of venous thrombosis (46). PAI-2 was originally discovered as an effective inhibitor of uPA activity in *in vitro* assays (140), however, compared with PAI-1, it is found to be a slower inhibitor of uPA by a factor of 10-fold and tPA by a factor of 50-fold in *in vitro* assays using recombinant proteins. PAI-2 is *one* of the most abundantly induced proteins in monocytes and macrophages in response to inflammatory stimuli, with induction reported over 105-fold (141), and multiple lines of evidence link PAI-2 to inflammatory pathways that sculpt the nature of innate immune responses [reviewed in (142)]. PAI-2 is found predominantly as an intracellular protein which is characterized by the lack of a classical secretory signal (143), and many of its immune modulatory activities are independent of inhibition of extracellular uPA (144, 145). Unlike PAI-1 deficiency, PAI-2 gene-deficient mice do not display any overt baseline changes in fibrinolysis or spontaneous thrombosis (146). It has been shown that in the stasis model of DVT, genetic deficiency of PAI-2 in mice significantly accelerates venous thrombus resolution, while thrombus formation is unaffected (46). This outcome was independent of any effect on the initial thrombus formation. The accelerated thrombus resolution was accompanied by increased levels of active uPA in PAI-2 deficient thrombi, with no significant effect on MMP-2 or−9 activities (46). While the increased uPA activity in the absence of PAI-2 seemingly suggested a direct role for PAI-2 in the inhibition of uPA, the mechanism appears more complex since the thrombi in PAI-2 deficient mice also had a concomitant reduction in PAI-1 levels, which could contribute to increased active uPA found in the thrombus. It was also found that genetic deficiency of PAI-1 significantly accelerated venous thrombus resolution similar to PAI-2 deficiency, but there were also substantial differences, since PAI-1 deficiency had an additional negative effect on thrombus formation and also altered intrathrombus MMP activities (46). Additional differences were observed in the repertoire of inflammatory cells present in venous thrombi between PAI-2 and PAI-1 deficient mice. Increased early neutrophil accumulation and decreased late macrophage infiltration was associated with PAI-2 deficiency and not PAI-1 deficiency. These data suggest that PAI-2 and PAI-1 modulate several distinct, but possibly overlapping pathways during venous thrombus resolution.

## Immune Cells in DVT Resolution

Experimental rodent models of DVT have revealed important insights into the innate immune cells and coordinated inflammatory processes involved in DVT and its resolution. Inflammation is central to both the initiation and resolution of venous thrombi and is directed at restoration of tissue integrity and function (147). Activation of the vein wall endothelium causes surface expression of cell adhesion molecules such as P and E-selectins that facilitate the transmigration of circulating leukocytes and microparticles (84, 148). As mentioned above, neutrophils, the most abundant immune cells, infiltrate the venous thrombus early and play a critical role during the early phase of venous thrombus resolution. They are found in both the vein wall and thrombus and are essential for initiating lysis of the thrombus via fibrinolysis and collagenolysis (149–152). Depletion of neutrophils in several experimental rodent models results in impaired venous thrombus resolution, associated with larger thrombi as well as increased fibrosis (153, 154). Neutrophils also facilitate recruitment of monocytes into the thrombus and as the thrombus matures, macrophage numbers increase and eventually become the predominant inflammatory cells present in the thrombus (42, 155). Macrophages produce various chemokines, inflammatory cytokines and matrix-degrading proteases such as uPA and MMPs that promote fibrinolysis and the tissue remodeling required to eventually restore blood flow in the thrombosed vein (84). In studies of the effect of MMP-9 deficiency on stasis DVT, it was found that MMP-9 modulates midterm vein wall collagen content, with an altered local inflammatory and profibrotic environment, likely directed by monocytes (40, 156). As potent phagocytic cells, macrophages also contribute to clearance of apoptotic neutrophils and other proteins within the thrombus.

Macrophages are present as a heterogenous population and based on *in vitro* studies, may be distinguished by two main polarization phenotypes: (1) those that promote inflammatory responses (M1-like or classically activated) which are induced by interferon-γ (IFN-γ) together with a variety of TLR agonists or by these agonists alone, and which express inflammatory mediators, such as TNF-alpha, IL-6, IL-12, and iNOS; and (2) those that attenuate inflammatory responses (alternatively activated M2-like) which express mediators such as Arginase-1, the mannose receptor (CD206) and the transcription factor Fizz1 (157). The role of macrophage polarization in venous thrombus resolution is only now emerging. Using the stasis model of venous thrombosis and resolution in mice, genetic deficiency or pharmacologic inhibition of p53 was shown to impair thrombus resolution and was associated with increased fibrosis and altered expression of MMP-2 (47). Using mice that lacked p53 in the myeloid cells, it was shown that the effect of p53 loss was mediated by cells of the myeloid lineage, resulting in enhanced polarization of the cytokine milieu toward an M1-like phenotype. In stasis (chronic) and non-stasis (acute and chronic) models of DVT resolution, a predominance of anti-inflammatory M2-like macrophages were identified in venous thrombi (158). Since CD206 positive M2-like macrophages play a critical role in mediating collagen turnover (87), a key event in the inflammatory vascular remodeling processes associated with venous thrombus resolution (84), M2 polarization is likely to be important for VTE resolution.

In addition to innate immune responses, there is evidence for adaptive immune regulation of sterile inflammation in DVT resolution. CD4^+^ and CD8^+^ T cells infiltrate the thrombus and vein wall rapidly on DVT induction and remain in the tissue throughout thrombus resolution (159). In the vein wall, recruited T cells were found to largely consist of effector-memory T (T_EM_) cells. Reducing the number of T_EM_ cells through a depletion recovery procedure showed that intravenous T_EM_ activation modulated neutrophil and monocyte recruitment and delayed thrombus neovascularization and resolution (159).

## Inflammatory Factors and Venous Thrombus Resolution

The process of venous thrombus resolution is associated with a number of changes in the expression of inflammatory cytokines (84), although there are only a few reports demonstrating a direct role for inflammatory cytokines in modulating the resolution of venous thrombi. Clinical studies show that the levels of serum cytokines including C-reactive protein, IL-6, IL-8, and TNF-alpha, are associated with the risk of VTE [(160–164) and reviewed in (165)]. IL-6 has been linked to fibrosis and it has been found that neutralization of IL-6 by systemic injection of antibodies in a stasis DVT model, accelerates thrombus resolution along with reducing monocyte recruitment and decreasing vein wall fibrosis (166). Global genetic deletion of IFN-gamma in mice was found to accelerate venous thrombus resolution through enhanced MMP-9 and VEGF expression (43). The TNF-alpha/TNF-receptor-rp55 signaling axis was also demonstrated to modulate venous thrombus resolution. Genetic deletion of the TNF-receptor-rp55 inhibited venous thrombus resolution and administration of an anti-TNF-alpha antibody or the TNF-alpha inhibitor (etanercept) had a similar effect (167). The mechanisms involved were determined to involve regulation of intrathrombic uPA, MMP-2, and MMP-9 levels (167). Direct administration of macrophage chemoattractant protein 1 (MCP-1) into experimental venous thrombi in a rat stenosis model stimulated increased thrombus resolution, which resulted in thrombus recanalization, independent of an effect on monocyte recruitment (168).

## Preclinical Studies of Modulators of DVT and Its Resolution

The majority of DVT studies focus on the contribution of various factors to the development (or initiation) of the venous thrombus, whereas the number of studies devoted to identifying modulators of venous thrombus resolution are limited. Listed in Table 1 are transgenic and other challenge mouse models that have revealed insights into mechanistic processes of venous thrombus resolution along with the effects on the fibrinolytic system.

**Table 1:** Effect of interventions in mouse models of deep vein thrombosis on thrombus resolution.

**Intervention**	**DVT model**	**Effect on venous thrombus resolution**	**Changes in the fibrinolytic system**	**References**
IFN-gamma gene deletion	Stasis	Enhanced	No changes in tPA, uPA, PAI-1 mRNA	(43)
p53 gene deletion	Stasis	Impaired	No change in active uPA	(47)
Tnfrp55 (tumor necrosis factor receptor p55) gene deletion	Stenosis	Impaired	Decreased uPA mRNA	(167)
Tbx21 (T-Box Transcription Factor TBX21) gene deletion	Stenosis	Enhanced	Decreased PAI-1 mRNA	(169)
Effector memory T cell depletion	Stenosis	Enhanced	Decreased tPA mRNA	(159)
Statin treatment	Stasis	Enhanced	Decreased PAI-1 protein	(170)
TLR9 gene deletion	Stasis	Impaired	N.D.	(171)
Activated Protein C treatment	Stasis	Enhanced	No changes in active uPA and PAI-1 protein	(45)
MMP-9 gene deletion	Stasis	Enhanced	Increased PAI-1 protein	(156)
MMP-2 gene deletion	Stasis	Impaired	N.D.	(172)
ApoE gene deletion	Stasis	Impaired	Reduced uPA and increased PAI-1 protein	(138)
Type 2 diabetes	Stenosis	Impaired	Reduced uPA and increased PAI-1 protein	(173)
CCR2 gene deletion	Stasis	Impaired	Reduced intra-thrombotic uPA positive cells	(42)
PAI-1 gene deletion	Stasis	Enhanced	Increased active uPA	(46)
PAI-2 gene deletion	Stasis	Enhanced	Increased active uPA and decreased PAI-1 protein	(46)
uPA gene deletion	Stenosis	Impaired	N.D.	(55)

*N.D., not determined*.

Although such models are instrumental in understanding disease pathophysiology, they are limited in terms of clinical applicability for the treatment of VTE. They also fail to simulate the clinical scenario in which patients usually present with an existing thrombus. On the other hand, outcomes from pharmacological modulation of preclinical DVT models provide more immediate promise for direct clinical application. Using a primate model of stasis induced venous thrombosis, it was found that prophylactic inhibition of P-selectin using a small molecule inhibitor (PSI-421) was effective in reducing the thrombus size, enhancing recanalization of the thrombus and reducing vein wall scarring, compared to treatment with low molecular weight heparin (Enoxaparin) (174). This observation was reconfirmed in a follow-up study using an anti-P-selectin aptamer (ARC5692) (175). When the p53 activator quinacrine was administered in a mouse model of stasis induced venous thrombosis, thrombus resolution was substantially accelerated and this was associated with a less intrathrombus inflammatory macrophage phenotype and reduced collagen deposition (47). Interestingly, quinacrine treatment also accelerated resolution of an existing thrombus, simulating the clinical scenario. Inhibition of IFN-gamma signaling by treatment with anti-IFN-gamma antibodies after the establishment of a stasis DVT in mice was effective in accelerating venous thrombus resolution, in addition to reducing fibrosis, without an effect on the coagulation function (43). Another study used anti-IL-6 antibodies to show that blockage of IL-6 resulted in reduced vein wall intima thickness and collagen deposition, although in this study the antibody was administered before the formation of the venous thrombus (166). In a rat stenosis model of venous thrombosis, a small molecule PAI-1 inhibitor (PAI-039; tiplaxtinin), accelerated venous thrombus resolution and increased vena cava blood flow at a low dose, although at a high dose paradoxically decreased venous thrombus resolution (176). These studies indicate that PAI-1 plays complex roles in this process.

## Current Therapies and Future Perspectives

There are between 350,000 and 600,000 cases per year of venous thromboembolism (VTE) in the U.S. and 100,000 deaths from PE (177, 178). DVT, a major cause of morbidity and mortality, has an incidence rate of 1 person per 1,000 annually (177). Anticoagulants are currently used for treatment of DVT, commonly either the vitamin K antagonist warfarin or direct-acting oral anticoagulants. In the case of warfarin, parenteral anticoagulation with low-molecular weight heparin is also prescribed for concomitant use. Direct-acting oral anticoagulants can be divided into two classes: direct thrombin inhibitors (dabigatran) and direct factor Xa inhibitors (apixaban, edoxaban, and rivaroxaban). Despite the use of anticoagulants, approximately 25 to 50% of DVT patients develop PTS and about 5% of patients suffering from an unresolved PE develop chronic thromboembolic pulmonary hypertension (CTEPH) as a late complication (179).

Surgical interventions for DVT are generally performed for large symptomatic lesions, particularly those that enlarge or worsen despite the anticoagulation therapy. These interventions focus on pharmacological thrombolytic therapy administered through a catheter positioned in or near the thrombus, as well as mechanical means to disrupt, aspirate or disperse the thrombus. Pharmacological therapy is most effective for acute DVT of <2 weeks duration. As the thrombus resolves into a more fibrotic lesion, the effectiveness of the pharmacological therapy diminishes. Invasive catheter-directed therapy for DVT is associated with multiple risks, including bleeding at the puncture site or in remote anatomic sites (such as brain). In general, these interventions for DVT cannot be used in patients with acute trauma, patients who have undergone recent surgery, patients with pregnancy and patients who are at risk for bleeding (180).

Invasive interventions for the treatment of DVT are often carried out with the intent to lower the future risk of PTS. A number of studies have demonstrated that early thrombolytic therapy of DVT results in less subsequent venous reflux, decreased symptoms of PTS and improved venous patency (181). A recent large multi-center trial (ATTRACT) with randomized patients with acute femoral or iliac DVT examined the efficacy of catheter-directed pharmacomechanical thrombectomy vs. standard anticoagulation plus compression stockings (182). The results showed no overall decrease in the rate of “mild-to-moderate” PTS, however there was a significant decrease in the incidence of acute pain and swelling and “moderate-to-severe” PTS at 2 years. While the scientific rationale for these results is not known, it seems that addressing thrombolysis by pharmacomechanical interventions are not sufficient to control PTS development. Thus, therapies that modulate the inflammatory response during venous thrombus resolution may be required to modulate the inflammation that promotes PTS.

## Author Contributions

SM and TA conceived and designed the manuscript. SM, TA, TJ, ND, MB, NP, and RS contributed to the literature review and writing of the paper. All of the authors read and approved the final manuscript.

### Conflict of Interest Statement

The authors declare that the research was conducted in the absence of any commercial or financial relationships that could be construed as a potential conflict of interest.
